# The bacterial pigment pyocyanin inhibits the NLRP3 inflammasome through intracellular reactive oxygen and nitrogen species

**DOI:** 10.1074/jbc.RA117.001105

**Published:** 2018-02-06

**Authors:** Sebastian Virreira Winter, Arturo Zychlinsky

**Affiliations:** From the Max Planck Institute for Infection Biology, Charitéplatz 1, 10117 Berlin, Germany

**Keywords:** inflammasome, inflammation, Pseudomonas, oxygen radicals, interleukin 1 (IL-1)

## Abstract

Inflammasomes are cytosolic complexes that mature and secrete the inflammatory cytokines interleukin 1β (IL-1β) and IL-18 and induce pyroptosis. The NLRP3 (NACHT, LRR, and PYD domains–containing protein 3) inflammasome detects many pathogen- and danger-associated molecular patterns, and reactive oxygen species (ROS)/reactive nitrogen species (RNS) have been implicated in its activation. The phenazine pyocyanin (PCN) is a virulence factor of *Pseudomonas aeruginosa* and generates superoxide in cells. Here we report that PCN inhibits IL-1β and IL-18 release and pyroptosis upon NLRP3 inflammasome activation in macrophages by preventing speck formation and Caspase-1 maturation. Of note, PCN did not regulate the AIM2 (absent in melanoma 2) or NLRC4 inflammasomes or tumor necrosis factor (TNF) secretion. Imaging of the fluorescent glutathione redox potential sensor Grx1-roGFP2 indicated that PCN provokes cytosolic and nuclear but not mitochondrial redox changes. PCN-induced intracellular ROS/RNS inhibited the NLRP3 inflammasome posttranslationally, and hydrogen peroxide or peroxynitrite alone were sufficient to block its activation. We propose that cytosolic ROS/RNS inhibit the NLRP3 inflammasome and that PCN's anti-inflammatory activity may help *P. aeruginosa* evade immune recognition.

## Introduction

Inflammation is an essential response to infection or tissue damage. Pattern recognition receptors detect infection or injury and initiate a process that culminates in cytokine secretion to orchestrate the recruitment of immune cells to the inflammatory site. Inflammasomes are cytosolic multiprotein complexes that regulate the secretion of the inflammatory cytokines IL-1β^2^ and IL-18 and a type of cell death called pyroptosis. Multiple pattern recognition receptors engage the assembly of inflammasomes, including the NOD- and AIM2-like receptors. All inflammasomes contain Caspase-1 and many engage the adapter protein apoptosis-associated speck-like protein containing a CARD (ASC). A well studied sensor is NACHT, LRR, and PYD domains–containing protein 3 (NLRP3), which is central in host microbial defense and in the pathogenesis of many diseases like atherosclerosis, diabetes, and Alzheimer's disease (1).

Macrophages require two steps to activate the NLRP3 inflammasome: transcriptional priming and a stimulus triggering inflammasome assembly. Many molecules activate the NLRP3 inflammasome, although it remains elusive how these chemically diverse molecules are recognized. It was proposed that NLRP3 is an ion sensor because most stimuli trigger potassium efflux (2). An alternative model posits reactive oxygen species (ROS) and nitrogen species (RNS) as the common NLRP3 inflammasome trigger (3, 4). Indeed, pharmacological or RNAi-based inhibition of the NADPH oxidase NOX2 blocks the NLRP3 inflammasome (3, 4). However, mice and patients with mutant NOX2 show normal or even increased NLRP3 inflammasome activation (4). Regardless, redox mechanisms regulate transcription of IL-1β and NLPR3 as well as IL-1β secretion and Caspase-1 activity (4). Moreover, mitochondrial ROS are essential for NLRP3 activation (5).

Nigericin, ATP, and crystalline substances such as silica are frequently used NLRP3 activators. Nigericin is a bacterial H^+/^K^+^ antiporter, ATP released by dead cells is sensed by P2X purinoceptor 7, and crystals destabilize the phagosome (1). All of these stimuli trigger NLRP3 inflammasome assembly by inducing K^+^ efflux (2). The NLRC4 inflammasome detects flagellin and the type III secretion system and is essential to sense pathogenic bacteria. The AIM2 inflammasome detects cytosolic double-stranded DNA and senses intracellular microbes.

The opportunistic pathogen *Pseudomonas aeruginosa* causes nosocomial infections and attacks immunocompromised hosts. This microbe has multiple pathogenic mechanisms that contribute to pathogenesis, including the blue pigment pyocyanin (PCN) (6). PCN is a membrane-permeable secreted phenazine that produces ROS/RNS and is an essential virulence factor (7, 8). It mainly produces superoxide anions by non-enzymatic transfer of electrons from NADH and NADPH to oxygen (9). PCN is exclusively produced by *P. aeruginosa* and is anti-bacterial against other microbes, whereas *Pseudomonas* strains are resistant to the phenazine (10). PCN also regulates iron uptake, redox homeostasis, and biofilm formation (7, 9). In the host, this phenazine inhibits cell growth and ciliary movements and induces cell death and cytokine release (7, 9, 11, 12). Notably, PCN oxidizes GSH, the most abundant thiol in cells and an important antioxidant. *P. aeruginosa* also secretes other phenazines, like 1-hydroxy-phenazine (1-HP) and the PCN precursor phenazine-1-carboxylic acid (PCA).

Here we show for the first time that PCN-dependent redox changes block the NLRP3 inflammasome in bone marrow–derived macrophages (BMDMs). PCN inhibits NLRP3 posttranslationally and ROS/RNS-dependently and acts upstream of speck formation and Caspase-1 maturation. We demonstrate a previously unrecognized anti-inflammatory effect of PCN and show that excessive ROS/RNS production silences the NLRP3 inflammasome.

## Results

### PCN blocks NLRP3-dependent Caspase-1 maturation and IL-1β release

**Figure 1.**
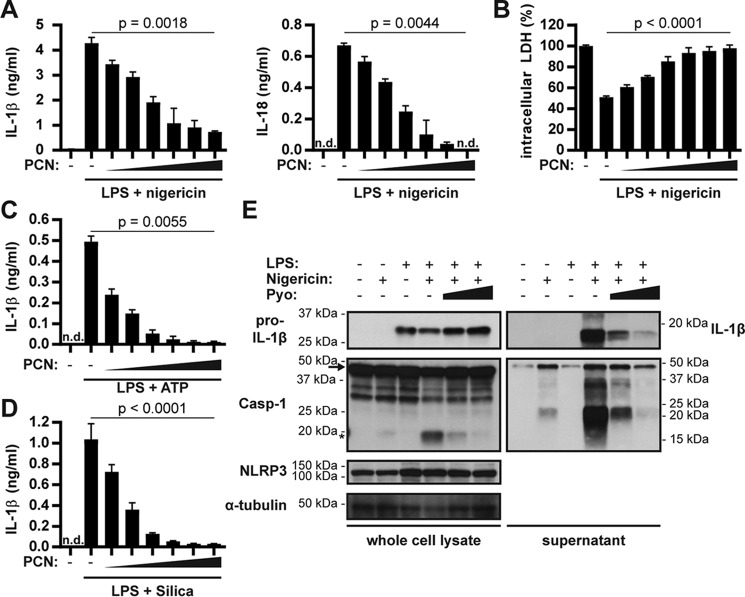
**Pyocyanin blocks Caspase-1 maturation and IL-1β release in response to NLRP3 activation.** *A–D*, BMDMs were first primed for 3 h with LPS and then incubated with 0, 10, 20, 40, 60, 80, or 100 μm PCN for 15 min before activating NLRP3 with nigericin (*A* and *B*) or ATP (*C*) for 1 h or silica (*D*) for 2 h. We quantified IL-1β and IL-18 (ELISA) and pyroptosis (intracellular LDH content) at the indicated time. *E*, we pretreated LPS-primed BMDMs with 0, 40, or 100 μm PCN for 15 min before activating NLRP3 with nigericin for 45 min. We collected supernatants and prepared whole-cell lysates 45 min after activation for Western blotting. *Arrows* indicate pro-Caspase 1 and *asterisks* the active p20 fragment. The graphs show mean ± S.D. from one representative experiment of three independent replicates. The Western blot is from one representative experiment of two independent replicates. Repeated measures one-way ANOVA was performed on three independent experiments for statistical analysis. *Pyo*, pyocyanin.

We examined whether PCN interferes with inflammasome activation by nigericin at concentrations it has in the sputum of cystic fibrosis patients (13). A short preincubation of LPS-primed BMDMs with PCN strongly reduced secretion of both IL-1β and IL-18 in a concentration-dependent manner (Fig. 1*A*). We also quantified intracellular lactate dehydrogenase (LDH) as a proxy for pyroptosis. As expected, NLRP3 activation reduced the LDH content to 50% compared with untreated cells (Fig. 1*B*). Notably, PCN rescued the cells from pyroptosis, as shown by the retention of intracellular LDH. PCN also inhibited IL-1β release in response to ATP (Fig. 1*C*) and silica (Fig. 1*D*). Importantly, PCN inhibited Caspase-1 processing and maturation of IL-1β, both hallmarks of inflammasome activation (Fig. 1*E*). PCN did not affect the levels of pro-IL-1β or NLRP3. These data show that PCN inhibits Caspase-1 maturation and the release of IL-1β and IL-18 in response to various NLRP3 inflammasome activators.

### PCN inhibits the NLRP3 inflammasome specifically

**Figure 2.**
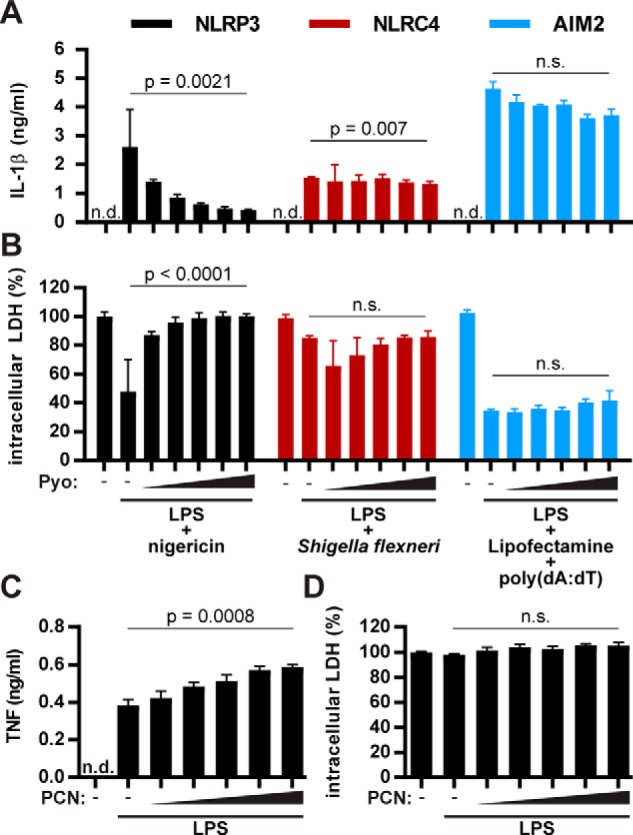
**Pyocyanin inhibits activation the NLRP3 inflammasome specifically.** *A* and *B*, LPS-primed BMDMs were treated with 0, 20, 40, 60, 80, or 100 μm PCN for 15 min before activating NLRP3 with nigericin for 1 h, NLRC4 by infection with *S. flexneri* for 1 h, and AIM2 by transfection of poly(dA:dT) and incubation for 2 h. We quantified IL-1β (ELISA) (*A*) and pyroptosis (intracellular LDH contents) (*B*) after the indicated times. *C* and *D*, BMDMs were first pretreated with 0, 20, 40, 60, 80, or 100 μm PCN for 15 min and then stimulated with LPS for 2 h. We quantified TNF (ELISA) (*C*) and pyroptosis (intracellular LDH content) (*D*) after 2 h. The graphs show mean ± S.D. from one representative experiment of three (*A* and *B*) or four (*C* and *D*) independent replicates. Repeated measures one-way ANOVA was performed on three (*A* and *B*) or four (*C* and *D*) independent experiments for statistical analysis. *n.s.*, not significant.

Because Caspase-1 and ASC are common to multiple inflammasomes, we tested which of these are blocked by PCN. We infected BMDMs with *Shigella flexneri* to activate NLRC4 or transfected the cells with double-stranded DNA to activate AIM2. PCN had almost no effect on IL-1β secretion after *S. flexneri* infection but led to minimal but significant inhibition of NLRC4 when used at the highest concentration of 100 μm. In contrast, PCN reduced AIM2 activation in response to cytosolic DNA mildly but not significantly (Fig. 2*A*). Furthermore, PCN did not rescue cells from NLRC4- or AIM2-dependent pyroptosis (Fig. 2*B*).

PCN did not block, but even slightly increased, TNF secretion upon LPS stimulation of BMDMs, suggesting that PCN is not a general inhibitor of cytokine production (Fig. 2*C*). Even at high concentrations, PCN was not toxic, as shown by the retention of intracellular LDH (Fig. 2*D*). Together, these data show that PCN interferes specifically with the NLRP3 inflammasome.

### PCN prevents inflammasome speck formation posttranslationally

**Figure 3.**
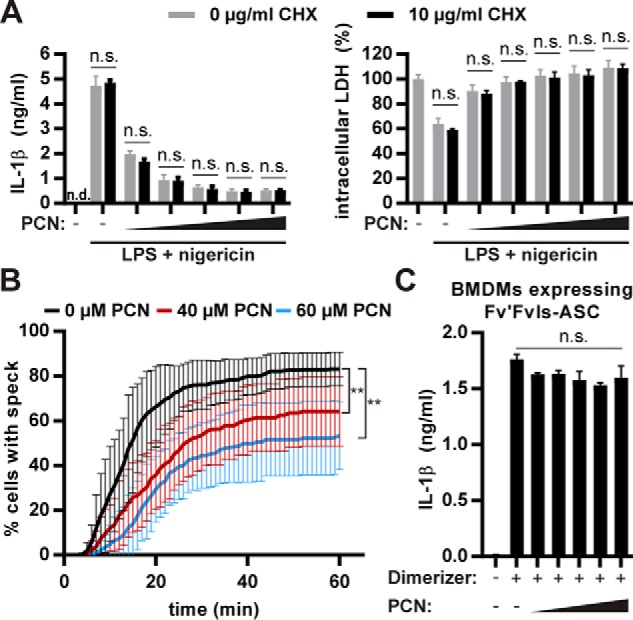
**Pyocyanin prevents inflammasome speck formation posttranslationally.** *A*, we first pretreated LPS-primed BMDMs with the indicated concentrations of the translation inhibitor CHX for 15 min and then incubated them with 0, 20, 40, 60, 80, or 100 μm of PCN for another 15 min. Subsequently, we activated NLRP3 with nigericin for 1 h and quantified IL-1β (ELISA) and pyroptosis (intracellular LDH contents). Repeated measures two-way ANOVA with Bonferroni's multiple comparison test was performed on three independent experiments for statistical analysis. *B*, we treated LPS-primed BMDMs expressing ASC-Grx1-roGFP2 with 0, 40, or 60 μm of PCN for 15 min. Then we activated NLRP3 with nigericin and quantified speck formation microscopically by imaging once a minute for 1 h. We calculated the percentage of cells showing a speck and used two-way repeated measures ANOVA with correction for multiple comparisons test (Dunnett) for statistical analysis. The corrected *p* values are 0.0079 for 0 *versus* 40 μm PCN and 0.0012 for 0 *versus* 60 μm PCN. *C*, we treated LPS-primed BMDMs expressing Fv'Fvls-ASC with 0, 20, 40, 60, 80, or 100 μm PCN for 15 min and induced speck formation with 500 nm B/B homodimerizer (AP20187). Repeated measures one-way ANOVA was performed on three independent experiments for statistical analysis. The graphs in *A* and *C* show means + S.D. and are from one representative experiment of three independent replicates. The graph in *B* shows means + S.D. from four independent experiments. *n.s.*, not significant.

PCN does not interfere with transcription or translation in our setting because it did not affect TNF secretion or IL-1β and NLRP3 levels. It is important to stress that, in the experiments presented here, we first primed the macrophages with LPS to trigger all required transcriptional responses before we added pyocyanin for only a short timeframe to focus on posttranslational effects of PCN. Nevertheless, we determined the effect of PCN on the mRNA levels of diverse pro-inflammatory cytokines in BMDMs. PCN did not influence cytokine transcription, irrespective of whether it was added after (Fig. S1*A*) or before (Fig. S1*B*) LPS-mediated priming. To confirm that PCN blocks NLRP3 activation posttranslationally, we show that the protein synthesis inhibitor cycloheximide (CHX) did not affect the inhibition of IL-1β secretion and pyroptosis (Fig. 3*A*). CHX was effective because it ablated TNF release in response to LPS (Fig. S2*A*).

Speck formation is a hallmark of NLRP3 inflammasome activation in response to classical activators such as nigericin and occurs upstream of Caspase-1 processing. To observe speck formation, we expressed a fusion protein of ASC and the glutathione redox potential sensor Grx1-roGFP2, which is a variant of the redox-sensitive green fluorescent protein roGFP2 and reversibly changes its fluorescence properties upon glutathione oxidation (Fig. S2*B*) (14). PCN significantly and dose-dependently reduced the percentage of cells with a visible speck in response to nigericin, suggesting that this compound interferes either with the interaction of NLRP3 with ASC or an upstream event (Fig. 3*B*).

To confirm that the effect of PCN was upstream of speck formation, we quantified IL-1β release upon artificial ASC aggregation. Fusions of caspases with the peptidyl-prolyl cis–trans isomerase FKBP12 induce cell death upon addition of a chemical dimerizer that has two FKBP12-binding domains (15). Analogously, we fused two copies of FKBP12_V36_ to ASC to generate Fv'Fvls-ASC, which dimerizes upon addition of the synthetic, cell-permeable ligand AP20187. We expressed this fusion protein in BMDMs and showed that chemically triggered dimerization of ASC results in IL-1β release (Fig. 3*C*). Notably, PCN did not interfere with IL-1β release and pyroptosis upon artificial ASC dimerization (Fig. S2*C*). These data suggest that PCN inhibits events upstream of speck formation.

### Pyocyanin inhibits the NLRP3 inflammasome through the production of ROS/RNS

**Figure 4.**
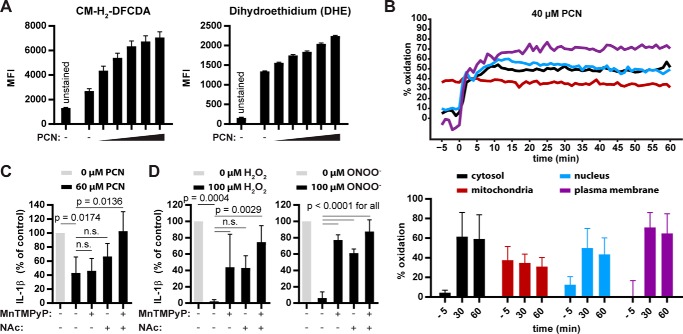
**Pyocyanin inhibits the NLRP3 inflammasome through the production of ROS/RNS.** *A*, we loaded BMDMs with 5 μm CM-H_2_-DCFDA or DHE (oxidation-sensitive dyes) before incubating them with 0, 20, 40, 60, 80, or 100 μm PCN for 30 min. We quantified the fluorescence by flow cytometry. *MFI*, mean fluorescence intensity. *B*, we treated BMDMs expressing Grx1-roGFP2 in the cytosol (*black*), the mitochondria (*red*), the nucleus (*blue*), or at the plasma membrane (*orange*) with 40 μm PCN and recorded the sensor fluorescence. We calculated the percentage of oxidized sensor by determining the dynamic range at the end of the stimulation. Graphs indicate the median oxidation of all cells analyzed for 1 h. Bar graphs indicate the mean oxidation of Grx1-roGFP2 + S.D. at the indicated time points. *C* and *D*, we incubated LPS-primed BMDMs with MnTMPyP or NAc for 5 min. Then we introduced ROS/RNS by adding PCN, hydrogen peroxide, or peroxynitrite for 15 min and activated NLRP3 with nigericin for 1 h. The graphs in *A* show geometric mean ± S.E. from one representative experiment of three independent replicates. The graphs in *B* show mean ± S.D. from one representative experiment of three independent replicates. The graphs in *C* and *D* show mean ± S.D. from three independent experiments. Repeated measures one-way ANOVA with Bonferroni's multiple comparison test was performed on three independent experiments for statistical analysis. *n.s.*, not significant.

We exposed BMDMs loaded with the ROS/RNS-detecting dyes CM-H_2_-DCFDA or dihydroethidium (DHE) to increasing PCN concentrations to confirm that PCN, like in other cells, causes ROS/RNS production in macrophages (Fig. 4*A*) (7). CM-H_2_-DCFDA preferentially senses peroxynitrite and the hydroxyl radical, whereas DHE primarily detects superoxide. Oxidation of both dyes indicated that PCN induced various ROS/RNS types. Besides PCN, 1-HP and, to a lesser extent, also PCA, but not phenazine, induced intracellular ROS (Fig. S3*A*).

To determine which intracellular sites are oxidized by PCN, we targeted the genetically encoded GSH redox potential sensor Grx1-roGFP2 to the cytosol, the nucleus, the mitochondria, or the plasma membrane of BMDMs by fusing it with the nuclear localization signal from SV40, the mitochondrial localization signal from ATP synthase protein 9 from *Neurospora crassa* or the C-terminal C*AAX* domain of human KRas, respectively. PCN oxidized GSH in the cytosol, the nucleus, and at the plasma membrane but, surprisingly, not in mitochondria, as quantified by time-lapse microscopy and ratiometric analysis (Fig. 4*B*). Representative images of the ratiometric analysis at 0 or 30 min after PCN treatment are shown in Fig. S4*A*. We also quantified the mitochondrial glutathione redox potential reported to be increased in response to nigericin challenge, a strong NLRP3 activator (16–20). Surprisingly, nigericin decreased mitochondrial GSH oxidation (Fig. S4*B*).

Finally, we pretreated BMDMs with two cell-permeable ROS/RNS scavengers: the cysteine-containing *N*-acetyl-l-cysteine (NAc) and the porphyrin MnTMPyP. NAc scavenges ROS/RNS and replenishes the intracellular GSH pool, whereas MnTMPyP is a superoxide dismutase mimetic. MnTMPyP also scavenges peroxynitrite and, under reducing conditions, also nitric oxide (NO) (21). Neither of the scavengers alone prevented the inhibitory effect of PCN on IL-1β release (Fig. 4*C*). In combination, however, NAc and MnTMPyP abrogated PCN's effect. Furthermore, ROS/RNS are sufficient to interfere with NLRP3 activation because both hydrogen peroxide (H_2_O_2_) and peroxynitrite (ONOO^−^) efficiently inhibited IL-1β secretion in response to nigericin, and addition of NAc or MnTMPyP rescued the cytokine release (Fig. 4*D*). In line with a requirement for ROS/RNS to inhibit NLRP3, 1-HP, and, to a lesser extent, PCA, but not phenazine, blocked the NLRP3 inflammasome (Fig. S3*B*). The data show that phenazines inhibit NLRP3 activation by producing intracellular ROS/RNS. To investigate whether NLRP3 itself may be reversibly oxidized, we differentially alkylated cysteines in BMDM lysates with the cysteine-reactive isobaric tag iodoTMT and found that, among the six cysteines of NLRP3 analyzed, several showed increased oxidation upon treatment with PCN (Fig. S5).

## Discussion

We show that PCN specifically blocked activation of the NLRP3 inflammasome upstream of speck formation. PCN did not interfere with conventional secretion or the activation of the NLRC4 or AIM2 inflammasomes. In contrast to the current view that ROS/RNS can activate NLRP3 (1), excessive oxidation by PCN inhibits this inflammasome. Our data show that PCN inhibits NLRP3 through intracellular ROS/RNS and GSH oxidation in compartments other than mitochondria. Moreover, intracellular ROS/RNS were sufficient to inhibit activation of NLRP3. Our data suggest that PCN does not interfere with events downstream of sensor activation because inflammasome signaling merges at ASC, and IL-1β release by artificial ASC dimerization remained unaffected (1).

Our findings demonstrate an unanticipated anti-inflammatory potential of PCN. PCN may also be anti-inflammatory by inducing neutrophil apoptosis and inhibiting the NADPH oxidase (7, 9). During the preparation of this manuscript, a study reported that PCN inhibits activation of the NLRP3 and NLRC4 inflammasomes in response to quorum sensing–deficient *P. aeruginosa* (22). However, this study did not investigate the contribution of ROS/RNS. Interestingly, PCN can also be pro-inflammatory by inducing oxidative stress, cytokine transcription, and the formation of neutrophil extracellular traps (7, 9, 12). Although short-term PCN treatment inhibits NLRP3 via a posttranslational mechanism, long-term exposure to oxidative stress may also influence transcription of inflammasome components or inflammatory cytokines (23). This pleiotropic effect reflects the complex physiology of ROS/RNS.

In contrast to previous studies in epithelial cells, lymphocytes, or neutrophils (24–27), PCN did not oxidize GSH in mitochondria. The observation that PCN-dependent oxidation of the cytosol in macrophages inhibits NLRP3 activation is in line with previous reports showing that increased superoxide content caused by a deficiency in cytosolic SOD1 blocks the NLRP3 inflammasome (1, 3–5, 28). The finding that the transcription factor Nrf2, which is an important mediator of the cellular antioxidant defense, is required for NLRP3 activation further underlines that excessive oxidation inhibits this inflammasome (29). Although we show that oxidative stress prevents NLRP3 inflammasome activation, this does not contradict previous studies showing that low levels of localized ROS/RNS, in particular within mitochondria, may trigger its activation. Thus, the subcellular localization of redox changes is crucial for NLRP3 activation. Of note, multiple studies report that mitochondrial ROS/RNS trigger NLRP3 activation, whereas others show that oxidation occurs downstream of inflammasome activation as a result of membrane damage (16–20, 30). Because inflammasome activation and pyroptosis occur shortly after each other and at different time points in individual cells, bulk analysis of cells most likely includes already dead cells, which could explain the discrepancies in the conclusions regarding whether ROS/RNS occur up- or downstream of inflammasome activation. Furthermore, many studies reporting that mitochondrial ROS activate NLRP3 based their conclusions on the use of ROS scavengers or inducers, and they detected mitochondrial ROS only with the DHE-based superoxide indicator MitoSOX, which is enriched in mitochondria by the membrane potential. Nigericin is a K^+^/H^+^ antiporter and disrupts the pH difference between the mitochondrial matrix and the intermembrane space. Because of compensatory mechanisms, the mitochondrial membrane potential may increase, whereas superoxide production decreases in response to nigericin (31–35). Notably, the increased membrane potential may also result in increased cationic dye enrichment, which complicates interpretation of MitoSOX-based measurements and may explain the contradictory results (36).

Only the combination of NAc and MnTMPyP prevented the PCN-mediated NLRP3 inhibition, suggesting that NO is relevant. This is consistent with the inhibition of NLRP3 by nitrosylation (37). Alternatively, PCN could interfere with the ubiquitination or phosphorylation of inflammasome components, which regulate NLRP3 activation (37–40). Ubiquitin-modifying enzymes and protein–tyrosine phosphatases contain catalytic cysteines prone to thiol oxidation (41). Interestingly, PTPN22, which dephosphorylates NLRP3, has an intracellular disulfide bond that could be redox-regulated (42).

The molecular target of PCN-induced oxidation responsible for the inhibition of the NLRP3 inflammasome remains to be determined. However, we present some evidence that NLRP3 itself may be reversibly oxidized at individual cysteine residues, which could explain why the PCN-mediated oxidative stress specifically blocks NLRP3 but not other inflammasomes.

PCN is an essential virulence factor, and the concentrations of this phenazine negatively correlate with lung function in cystic fibrosis patients (8, 13, 43). Interestingly, PCN-deficient, but not PCN-competent, *P. aeruginosa* induce IL-1β during lung infection (43). *P. aeruginosa* is sensed by many innate immune mechanisms, including NLRC4 and NLRP3 inflammasomes, and secretes two exotoxins that interfere with Caspase-1 (44–46). However, depending on the model, inflammasomes can be protective or cause excessive tissue damage in *P. aeruginosa* infections (47).

Quorum sensing is important for *P. aeruginosa* virulence and induces PCN in biofilms (44). We speculate that *P. aeruginosa* uses quorum sensing to promote PCN secretion and evade detection by NLRP3. This may explain why biofilms of *P. aeruginosa* establish infections effectively.

## Experimental procedures

### ROS detection by flow cytometry

We loaded BMDMs with 5 μm 5-(and-6)-chloromethyl-2′,7′-dichlorodihydrofluorescein diacetate acetyl ester (CM-H2-DCFDA) or 5 μm DHE (both from Thermo Fisher Scientific) in serum-free RPMI medium for 20 min at 37 °C. Cells were washed and resuspended in RPMI medium with 10% FCS and PCN, phenazine (both from Sigma-Aldrich), 1-HP (abcr GmbH), or PCA (Apollo Scientific Ltd). After 30 min, cells were centrifuged and resuspended in 0.2% FCS/PBS containing 0.6 μm DRAQ7 (BioStatus) and analyzed with a MACSQuant Analyzer 10 flow cytometer. We analyzed the data with FlowJo v10 and excluded doublets and DRAQ7-positive cells.

### Cell culture and inflammasome activation

We differentiated BMDMs from C57BL/6 mice (The Jackson Laboratory) for 7 days with L929 cell–conditioned medium. ASC and Grx1-roGFP2 constructs were retrovirally transduced to BMDMs 72 h after isolation.

Inflammasomes were activated with 10 μm nigericin, 200 μg/ml silica (both from Sigma-Aldrich), or 3 mm ATP (GE Healthcare) (all NLRP3), an overnight culture of *S. flexneri* strain M90T at a multiplicity of infection of 20 (NLRC4), or transfection of poly(dA:dT) (Invivogen) using Lipofectamine 2000 (Thermo Fisher Scientific) (AIM2). The B/B homodimerizer (AP20187) was from Clontech and CHX from Sigma-Aldrich.

### LDH determination

We quantified LDH with the CytoTox 96 non-radioactive cytotoxicity assay (Promega), modified as follows. At the end of an experiment, the cells were washed and lysed with 1% IGEPAL CA-630/PBS to measure LDH. Untreated cells were used as a reference.

### ROS scavenging

We primed BMDMs with 100 ng/ml LPS (Enzo Life Sciences) for 3 h before adding either 10 mm NAc (Sigma-Aldrich), 200 μm MnTMPyP (Enzo Life Sciences), or both for 5 min. We added 60 μm PCN, 100 μm H_2_O_2_ (Sigma-Aldrich), or 100 μm peroxynitrite (Merck Millipore) for 15 min before activating NLRP3 with 10 μm nigericin.

### Cysteine oxidation assay

We transduced BMDMs during differentiation with a retrovirus to express FLAG-tagged mouse NLRP3. We primed differentiated BMDMs with 100 ng/ml LPS (Enzo Life Sciences) for 3 h before adding either 100 μm PCN or DMSO as a control. Cells were washed with PBS and lysed in 50 mm Bicine (pH 8.0) with 8 m urea, 1% IGEPAL CA-630, 5 mm EDTA, and one reagent of the iodoTMTsixplex (4 mm, Thermo Fisher Scientific) for 1 h. Residual free thiols were scavenged by addition of 10 mm iodoacetamide for 15 min, and proteins were precipitated with acetone at a final concentration of 80% for 24 h at −20 °C. We washed the protein pellets twice with 80% acetone and resuspended the proteins in 50 mm Bicine (pH 8.0) with 8 m urea, 10 mm EDTA, and 800 mm NaCl. Initially oxidized cysteines were reduced by 1 mm tris(2-carboxyethyl)phosphine for 10 min and then alkylated with another reagent of the iodoTMTsixplex (4 mm) for 1 h. FLAG-tagged mouse Nlrp3 was enriched by anti-FLAG M2 affinity–agarose gel for 30 min at 4 °C. The slurry was washed three times with 50 mm Bicine (pH 8.0) with 0.8 m urea, 1 mm EDTA, and 80 mm NaCl, and proteins were eluted from the beads with 0.1 m glycine (pH 3.5) and immediately mixed with 500 mm Bicine to neutralize the pH. We then digested the proteins with LysC and trypsin (Sigma-Aldrich) in the presence of 2 m urea for 24 h. Samples were then desalted by stage tipping and analyzed by MS/MS with an Q Exactive HF-X instrument (Thermo Fisher Scientific). Mass spectrometry data were processed with the MaxQuant software.

### Imaging

We imaged BMDMs in ibidi dishes in live-cell imaging solution (Thermo Fisher Scientific) supplemented with 2% FCS, 2 mm l-glutamine, 100 units/ml penicillin, 100 μg/ml streptomycin, 1× minimum Eagle's medium vitamin solution, and 4 g/liter d-glucose. We acquired images with a Leica TCS SP8 laser-scanning confocal microscope with a temperature chamber. We recorded Grx1-roGFP2 emission between 496 and 535 nm (excitation at 405 and 488 nm) and subtracted autofluorescence in response to 405-nm excitation. At the end of each experiment, we added 2 mm DTT and 16 mm H_2_O_2_ to fully reduce/oxidize the probe. We analyzed the images with Fiji Is Just ImageJ (Fiji) and excluded detached cells from the analysis. Ratios were normalized to percent oxidation as described previously (48).

### Cloning

We replaced the pgk-neo cassette in MSCVneo (Clontech Laboratories) with an IRES-GFP to generate GFP-RV. We excised the IRES-GFP from GFP-RV with EcoRI to generate RV2 and inserted a multiple cloning site (BglII, XhoI, NcoI, NotI, and EcoRI) between the original BglII/EcoRI sites. We amplified all constructs of Grx1-roGFP2 from pLPCX-Grx1-roGFP2, ASC from mouse cDNA, and Fv'Fvls from pSH1/Sn-E-Fv'Fvls-E. We generated the fusion constructs ASC-Grx1-roGFP2 and Fv'Fvls-ASC by overlapping PCRs. We cloned all sensors into RV2 at the NcoI/NotI sites and inserted the artificially dimerizable ASC fusion Fv'Fvls-ASC into GFP-RV using XhoI/SalI sites.

### Western blot analysis

We precipitated supernatants by mixing with methanol/chloroform and lysed BMDMs in 1% IGEPAL CA-630/PBS with cOmplete EDTA-free protease inhibitor (Roche). Mouse monoclonal IgG2b (Cryo-2) anti-NLRP3 and mouse monoclonal IgG1 (Casper-1) anti-Caspase-1 were from AdipoGen. Rabbit polyclonal anti-IL-1β was from Abcam, mouse monoclonal IgG1 (DM1A) anti-α-tubulin was from Sigma-Aldrich, and secondary antibodies were from Jackson ImmunoResearch Laboratories.

## Author contributions

S. V. W. and A. Z. conceptualization; S. V. W. data curation; S. V. W. formal analysis; S. V. W. validation; S. V. W. visualization; S. V. W. methodology; S. V. W. writing-original draft; A. Z. supervision; A. Z. project administration; A. Z. writing-review and editing.

## Supplementary Material

Supporting Information

## Footnotes

2 The abbreviations used are:

IL: interleukin
ASC: apoptosis-associated speck-like protein containing a CARD
ROS: reactive oxygen species
RNS: reactive nitrogen species
PCN: pyocyanin
1-HP: 1-hydroxy-phenazine
BMDM: bone marrow–derived macrophage
PCA: phenazine-1-carboxylic acid
LPS: lipopolysaccharide
LDH: lactate dehydrogenase
TNF: tumor necrosis factor
CHX: cycloheximide
CM-H_2_-DCFDA: 5-(and-6)-chloromethyl-2′,7′-dichlorodihydrofluorescein diacetate acetyl ester
DHE: dihydroethidium
NAc: *N*-acetyl-l-cysteine
NO: nitric oxide
FCS: fetal calf serum
Bicine: *N*,*N*-bis(2-hydroxyethyl)glycine
ANOVA: analysis of variance
IRES: internal ribosomal entry site.
