# Supplementary material for: The bacterial pigment pyocyanin inhibits the NLRP3 inflammasome through intracellular reactive oxygen and nitrogen species
Source: J Biol Chem. 2018 Feb 6;293(13):4893–900. doi: 10.1074/jbc.RA117.001105 (PMC5880120; doi:10.1074/jbc.RA117.001105)
Supplement: Supporting Information [file supp_293_13_4893__index.html]

The bacterial pigment pyocyanin inhibits the NLRP3 inflammasome through intracellular reactive oxygen and nitrogen species — Pyocyanin inhibits the NLRP3 inflammasome — Supporting Information 

# The bacterial pigment pyocyanin inhibits the NLRP3 inflammasome through intracellular reactive oxygen and nitrogen species

## Supporting Information

- Supplemental Data - Supplementary Figures S1 - S5
