# Supplementary material for: The bacterial pigment pyocyanin inhibits the NLRP3 inflammasome through intracellular reactive oxygen and nitrogen species
Source: J Biol Chem. 2018 Feb 6;293(13):4893–900. doi: 10.1074/jbc.RA117.001105 (PMC5880120; doi:10.1074/jbc.RA117.001105)
Supplement: Supporting Information [file supp_RA117.001105_134131_1_supp_69169_p3qtbq.pdf]

The bacterial pigment pyocyanin inhibits the NLRP3 inflammasome through intracellular ROS/RNS

Sebastian Virreira Winter & Arturo Zychlinsky

<u>Supplemental Data</u>

Supplementary Figure S1: S-2
Supplementary Figure S2: S-3
Supplementary Figure S3: S-4
Supplementary Figure S4: S-5
Supplementary Figure S5: S-6

Supplementary Figure S1

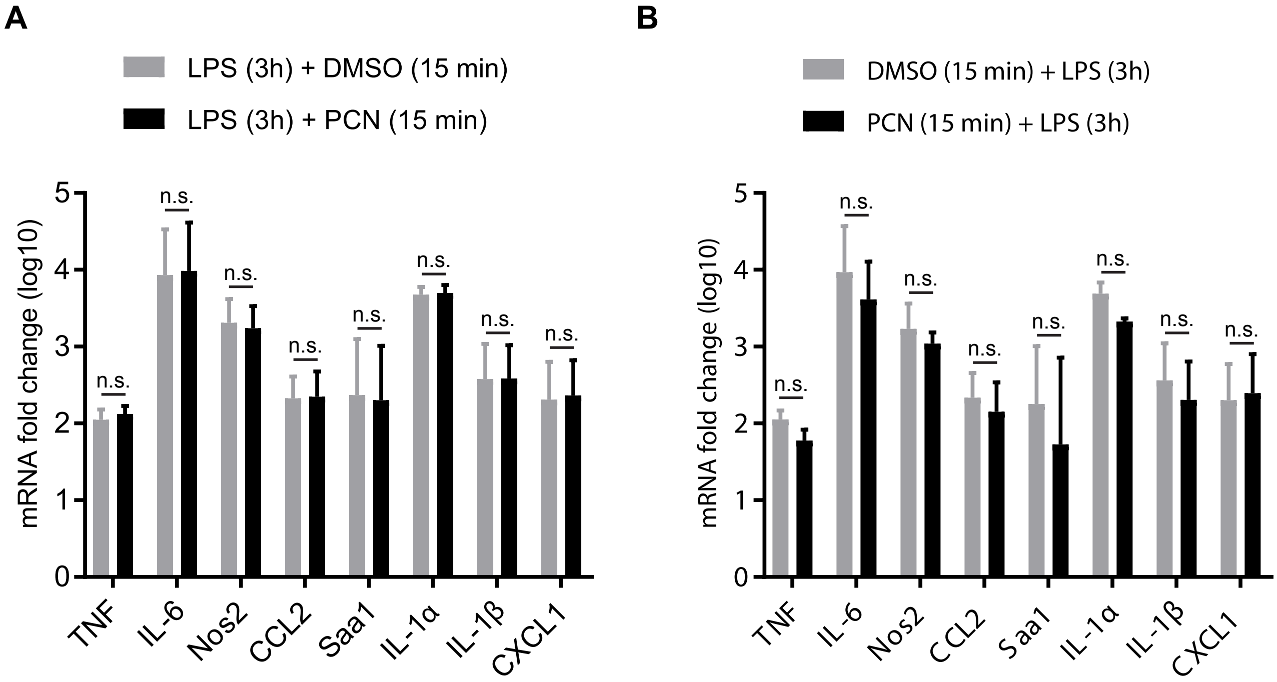

Supplementary Figure S1

(A) We first pretreated LPS-primed BMDMs with 100 ng/ml of LPS for three hours and then added DMSO as a control or 100 μM PCN for 15 min. We quantified mRNA expression levels of the indicated cytokines by qRT-PCR. The mRNA fold changes are in relation to the levels in naïve BMDMs. **(B)** We first treated BMDMs with DMSO or 100 μM PCN for 15 minutes and then primed the cells with 100 ng/ml LPS for three hours. The mRNA levels were determined as in (A). Graphs show means + SD from three independent replicates. A paired Student's t-test with Benjamini-Hochberg correction was performed for statistical analysis (significance cut-off was 0.05).

Supplementary Figure S2

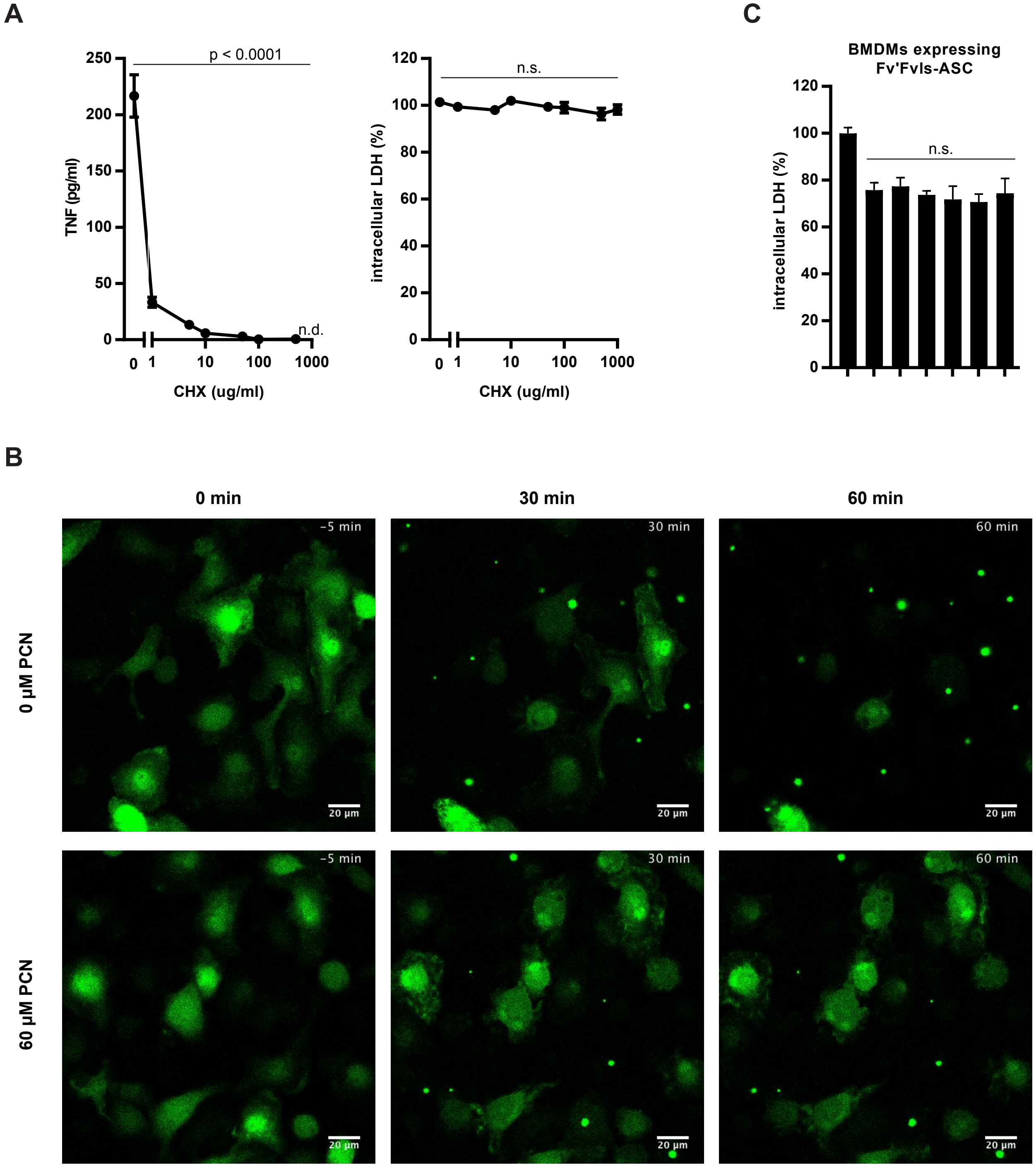

Supplementary Figure S2

(A) We first pretreated LPS-primed BMDMs with the indicated concentrations of the translation inhibitor cycloheximide (CHX) for 15 minutes, and then incubated them with 100 ng/ml of LPS. We quantified TNF release (ELISA) and pyroptosis (intracellular LDH content) after 2 hours. **(B)** We pretreated LPS-primed BMDMs expressing ASC−Grx1−roGFP2 with 0 μM or 60 μM PCN for 15 minutes before we activated NLRP3 by adding 10 μM nigericin. Speck formation was observed by time-lapse confocal microscopy. Cells were excited with a 488 nm laser and emission was recorded between 496-535 nm. **(C)** We treated LPS-primed BMDMs expressing Fv'Fvls−ASC with 0,20,40,60,80 or 100 μM PCN for 15 minutes and induced speck formation with the B/B homodimerizer (AP20187). Graphs in (A) & (C) show means + SD and are from one representative experiment of two independent replicates. Repeated measures one-way ANOVA on two independent replicates was performed for statistical analysis. Pictures in (B) are representatives from four independent experiments.

Supplementary Figure S3

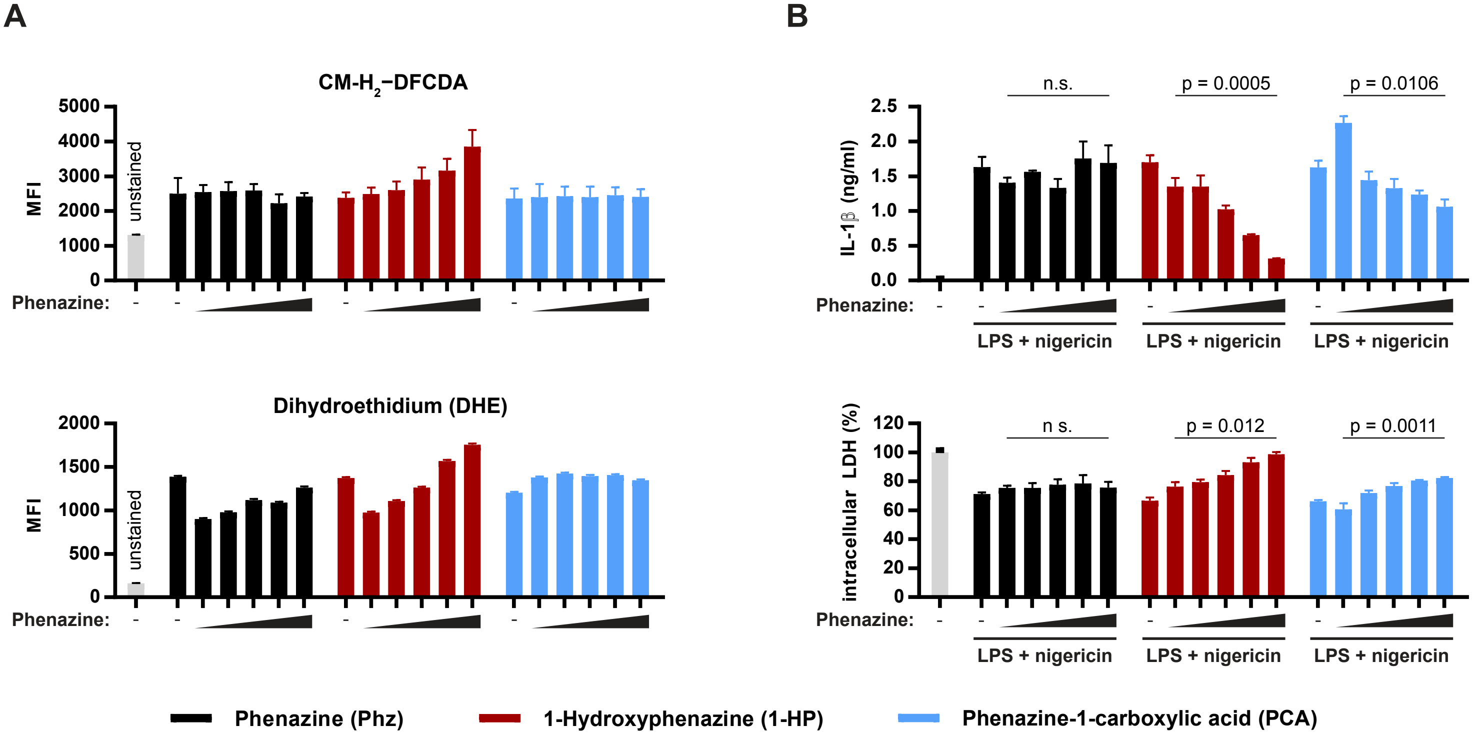

Supplementary Figure S3

(A) We loaded BMDMs with 5 µM of the oxidation-sensitive dyes CM−H2−DCFDA or Dihydroethidium (DHE) before incubating them with 0, 20, 40, 60, 80, or 100 µM of the indicated phenazine for 30 minutes. We quantified fluorescence intensities by flow cytometry. Graphs show geometric mean + SEM from one representative experiment of three independent replicates. **(B)** We pretreated LPS-primed BMDMs with 0, 20, 40, 60, 80 or 100 µM of the indicated phenazines for 15 minutes and activated NLRP3 with by adding 10 µM nigericin. We quantified IL-1β release (ELISA) and pyroptosis (intracellular LDH content) after 1 hour. Graphs show mean + SD from one representative experiment of three independent replicates. Repeated measures one-way ANOVA was performed on the three independent replicates for statistical analysis.

Supplementary Figure S4

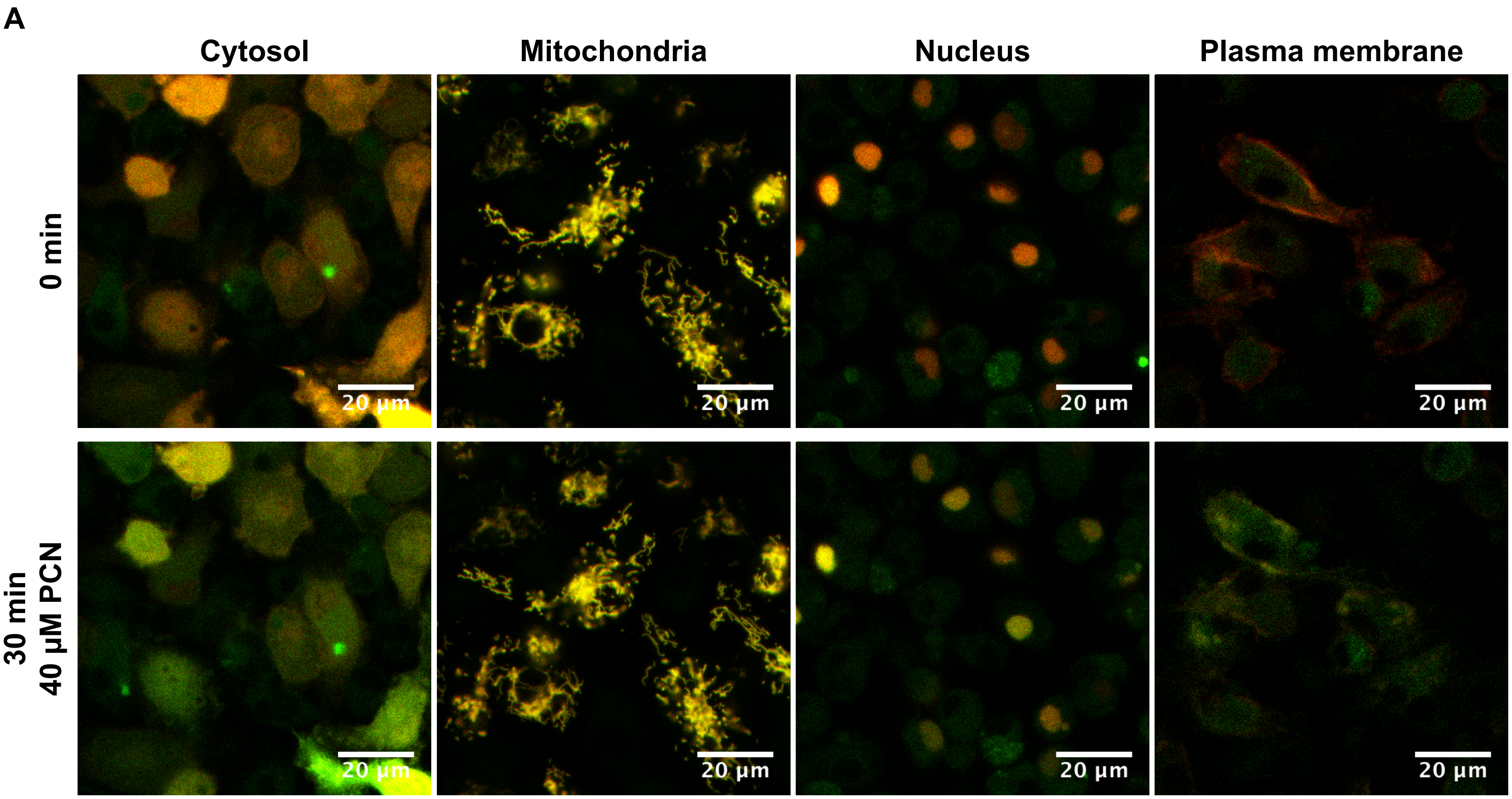

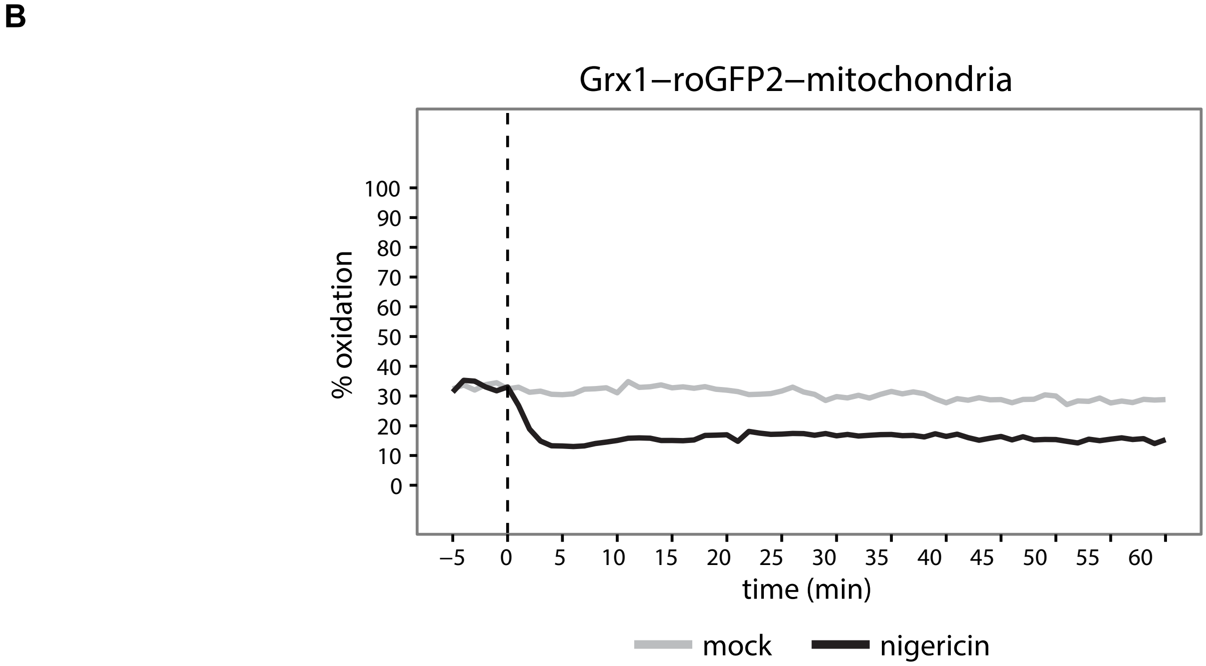

Supplementary Figure S4

(A) We treated BMDMs expressing Grx1-roGFP2 in the cytosol, the mitochondria, the nucleus or at the plasma membrane with 40 μM PCN and recorded the sensor fluorescence. Images show representative images just before (0 min) or 30 min after stimulation. The fluorescence signal in response to 405 nm excitation is false-colored in green and the fluorescence signal in response to 488 nM excitation is false-colored in red.
(**B**) We stimulated BMDMs expressing Grx1−roGFP2 within mitochondria with a vehicle control or 10 μM nigericin at timepoint 0 and recorded the sensor fluorescence. We calculated the percentage of oxidized sensor by determining the dynamic range at the end of the stimulation. Graphs indicate median oxidation of all cells analyzed for 1 hour.

Supplementary Figure S5

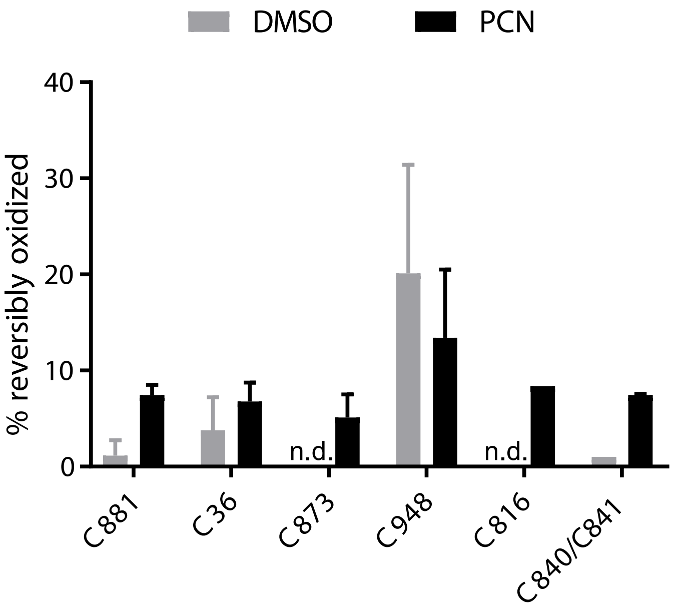

Supplementary Figure S5

BMDMs expressing FLAG-tagged NLRP3 were primed with 100 ng/ml LPS for three hours and then treated with 100 µM PCN or DMSO as a control for 15 minutes. Reduced and reversibly oxidized cysteines were differentially alkylated with the cysteine-reactive isobaric tag iodoTMT to determine the oxidation state of individual cysteines. The samples were digested with trypsin and peptides were analyzed by MS/MS. Graphs show mean + SD from individual MS/MS scans of two independent replicates. n.d. = not determined.
